# Breast Massage, Implant Displacement, and Prevention of Capsular Contracture After Breast Augmentation With Implants: A Review of the Literature

**Published:** 2017-12-21

**Authors:** Aditya Sood, Erica Y. Xue, Christopher Sangiovanni, Paul J. Therattil, Edward S. Lee

**Affiliations:** ^a^Department of Plastic and Reconstructive Surgery, The Ohio State University Medical Center, Columbus, OH; ^b^Division of Plastic and Reconstructive Surgery, Rutgers New Jersey Medical School, Newark, NJ

**Keywords:** Capsular contracture, breast implants, breast massage, Baker classification, implant displacement

## Abstract

**Objective:** Capsular contracture, the most common complication following breast augmentation with implants, is a complex inflammatory reaction that ultimately leads to fibrosis at the contact site between the implant and tissue. A number of peri-, pre-, and postoperative techniques have been postulated and implemented by many surgeons to reduce the incidence of capsular contracture. Breast massage and implant displacement technique is a commonly recommended practice that has not been well studied in regard to capsular contracture prevention. The authors present a review of the literature addressing methods and efficacy of massage and implant displacement techniques after breast augmentation. **Methods:** A literature review was performed using PubMed and the Cochrane Collaboration Library for primary research articles on breast massage or implant displacement after breast augmentation with implants for breast contracture prevention between January 1975 and March 2017. Exclusion criteria were studies that were focused on the treatment rather than prevention of breast contracture, addressed other strategies of preventing contracture as the main focus, or did not report the number of patients studied. Information related to massage technique and capsular contracture outcomes was extracted. **Results:** The literature search yielded 4 relevant studies, with a total of 587 patients. Outcomes evaluated included massage technique, onset of massage, frequency of massage, and incidence of capsular contracture. Breast massage was introduced between 2 days and 2 weeks postoperatively, performed twice daily, and lasted from 2 to 5 minutes for each breast. Final postoperative follow-up concluded between 6 and 36 months. The average capsular contracture rate was similar, 31% (range, 0-35) in the massage group versus 40% (range, 30-90) in the nonmassage group. **Conclusions:** While multiple techniques have been proposed and practiced in the prevention of capsular contracture, breast massage and implant displacement techniques remain controversial. While there is a method to measure adequacy of breast massage pressure, it is not widely utilized. The available data do not support breast massage to prevent capsular contracture; more studies with standardized techniques are needed to better assess the efficacy of breast massage in preventing capsular contracture.

Capsular contracture following breast augmentation with implants is a complex inflammatory reaction, ultimately leading to fibrosis at the contact site between the implant and tissue, followed by contracture of this tissue. It is the most common complication following breast augmentation and is a multifactorial and multicellular process. Capsular contracture is graded according to Baker's^1^ classification:

Grade I. The breast is normally soft and appears natural in size and shape.
Grade II. The breast is a little firm but appears normal.
Grade III. The breast is firm and appears abnormal.
Grade IV. The breast is hard, painful to touch, and appears abnormal.

The etiology of implant capsular contracture encompasses many patient-dependent, implant-specific, and polymicrobial factors. There are a number of pre-, intra-, and postoperative techniques that are implemented by many surgeons to prevent capsular contracture. Various breast massage and implant displacement techniques are believed to reduce the incidence of capsular contracture and are commonly recommended after breast augmentation. Some of these techniques include varying methods of displacement, manual pressure, and massaging of the implant into the cavity. However, the efficacy of these postoperative maneuvers has not been well studied. This literature review examines the relationship between the rate of capsular contracture and breast massage following augmentation mammaplasty with implants.

## METHODS

PubMed and Cochrane databases were searched by the authors from January 1975 through March 2017. In addition, bibliographies of each relevant citation were reviewed for additional sources. The following search terms were used as both subjects and key words: “breast contracture” AND (“spherical” OR “capsular”). The initial PubMed search yielded 1039 studies. The Cochrane database search yielded 82 studies. Two independent reviewers evaluated the titles and abstracts of all studies without language restrictions and subsequently included or excluded studies on the basis of the inclusion and exclusion criteria.

The authors included studies that were published in scientific journals and involved patients who underwent breast augmentation mammoplasty with implant placement and performed breast exercises for the purpose of preventing breast contracture. The authors excluded animal studies, studies that were focused on the treatment of breast contracture, studies that addressed other strategies of preventing contracture as the main focus, or studies that did not report the number of patients studied.

Manuscripts of abstracts that met criteria were reviewed as a second stage. The final pool comprised 4 studies with a total of 587 patients. Breast exercise and massage techniques were evaluated and compared, as was the proportion of patients who developed capsular contracture.

## RESULTS

### Literature Search

Electronic literature search yielded 4 studies that met the inclusion criteria. The outcomes evaluated included the number of patients, intraoperative methods, massage technique, onset and frequency of massage, and incidence of capsular contracture (Table 1). All 4 studies investigated capsular contracture rate with breast massage after augmentation mammaplasty with implants.

### Breast massage techniques, timing, and contracture rates

Vinnik^2^ studied the effects of intraoperative instillation of triamcinolone and postoperative capsule expansion exercises on the capsular contracture rate in 82 patients. For patients who received steroids, 40 mg of triamcinolone was instilled intraoperatively at the time of implant insertion. For patients who performed capsule expansion exercises, the exercises began 2 weeks postoperatively. The exercises consisted of 3 maneuvers performed twice daily in a supine position. During these maneuvers, the breast prosthesis was pressed firmly against the capsule in a uniform fashion for 15 seconds across the superior pole of the breast; each exercise session lasted less than 2 minutes. Patient follow-up ranged from 12 to 36 months postoperatively. Vinnik^2^ reported an overall spherical contracture (grades II-IV) rate of 50% (n = 41). Of the 27 patients who did not receive triamcinolone or capsule expansion exercises, 74% (n = 20) developed capsular contracture. Of 17 patients who received triamcinolone in each implant pocket but did not perform capsule expansion exercises, 58% (n = 10) developed capsular contracture. Of 38 patients who received both triamcinolone and capsule expansion exercise, 29% (n = 11) developed capsular contracture. The rate of capsular contracture was highest in the control group (patients who did not receive intraoperative steroids or capsule expansion exercises) and lowest in patients who received both intraoperative steroids and capsule expansion exercises.

In the second study, Hipps et al^3^ investigated the effect of capsule expansion exercises, intraoperative triamcinolone instillation, and drain placement on capsular contracture rates in 453 patients. However, it was unclear which patients received 60 mg of triamcinolone and/or drain placement in addition to the capsule expansion exercises; therefore, only the capsule expansion exercise results were reported. Patients began capsule expansion exercises 2 weeks postoperatively. The exercises were done “in the manner described by Vinnik,” twice daily, in all 3 directions, and for a total period of 2 minutes each.^2^^,^^3^ Patients were followed postoperatively at 1 week, 2 weeks, monthly for 3 months, every 3 months for 1 year, and annually after the first year. Hipps et al^3^ reported an overall capsular contracture (grades II-IV) rate of 32% (n = 147) after bilateral simple augmentation mammaplasty. Of the 210 patients who did not perform capsule expansion exercises, 30% (n = 62) developed capsular contracture. In 243 patients who performed capsule expansion exercises, 35% (n = 85) developed capsular contracture. The rate of capsular contracture was higher in patients who performed capsule expansion exercises compared with those who did not, although it must be noted that this difference was not statistically significant.

Riddle^4^ investigated age, prosthesis size, and capsule expansion exercises in 40 patients. Patients began capsule expansion exercises on the third postoperative day. Patients were evaluated at 1 week, 2 weeks, 1 month, 3 months, and 6 months postoperatively. The exercises were performed in the supine position, with multiple clockwise maneuvers exerting as much force as tolerated, for 5 minutes per breast, twice daily. Riddle's^4^ overall capsular contracture rate was 45% (n = 18). None of the 20 patients in the capsule expansion exercise group developed capsular contracture. In 20 patients who did not perform capsule expansion exercises, 90% (n = 18) developed capsular contractures (grades II-IV).^4^ All subjects reported following the exercise regimen between 1 and 3 times a day but did not time the exercise period for 5 minutes.^4^ There was no relationship between age and prosthesis size and the development of capsular contracture.

Finally, Becker and Prysi^5^ focused on capsular contracture rates in 12 women performing breast massage with adequate pressure, which was determined using a pressure manometer. Breast massages began on the second postoperative day. Massage technique was explained to the patient by the surgeon and the nurse, although the article did not describe the technique in detail, nor did it specify the frequency or duration of massage. At 4 weeks after operation, a pressure manometer was connected to a filling port to measure pressure applied to the implant. After physician demonstration of breast massage, patients repeated the massage while watching the pressure manometer. All patients generated adequate massage pressures between 80 and 300 mm Hg by 6 weeks postoperatively. Becker and Prysi^5^ reported 1 case out of 12 total patients (or 8%) of mild capsular contracture (grade not specified).

All 4 studies reported capsular contracture rates in the context of performing breast massage exercises. It is important to note that the studies differed in surgical technique, including incision site, antibiotic irrigation, steroid instillation, implant type, and location of the implant pocket. Specifically, Vinnik^2^ employed inframammary fold (IMF) incisions with smooth, gel-filled implants without fixation patches; he did not specify pocket plane. Hipps et al^3^ also used IMF incisions with smooth, gel-filled implants without fixation patches utilizing a retromammary (subglandular) pocket. Riddle's^4^ study specified a subpectoral insertion for double-lumen gel implants but did not specify the incision site. Becker and Prysi^5^ included procedures involving IMF, periareolar, or axillary incisions with an unspecified expander implant.

The studies also differed in massage technique, frequency of massage, as well as the timing of massage onset after surgery (Table 2). Breast massage was introduced between 2 days and 2 weeks postoperatively, performed twice daily in a supine position, and lasted from 2 to 5 minutes for each breast; final postoperative follow-up occurred between 6 and 36 months.

Of a total of 587 patients, 313 patients (53%) performed breast displacement massage or capsule expansion exercises and 274 (47%) did not perform any exercises after augmentation mammaplasty. In the breast massage group, the capsular contracture rate was 31% (n = 97) and ranged from 0% to 35%. In the nonmassage group, the capsular contracture rate was 40% (n = 110) and ranged from 30% to 90%. Because of variance in groups and limited study data, statistical analysis could not be performed.

## DISCUSSION

Following implant placement in breast augmentation, capsules of tightly woven collagen fibers form around the foreign body and wall the implant off. In capsular contracture, the physiologic fibroblast response becomes overreactive through interactions with inflammatory cells and extracellular matrix.^6^ Microbial biofilm formation is currently widely accepted as the cause of chronic inflammation and subsequent capsular contracture.^7^ Biofilm consists of bacteria enclosed within a matrix of their own excreted polysaccharides. The biofilm structure allows bacteria to densely adhere to prosthetic and biological surfaces while conferring resistance against antibiotics and host defenses.^7^^,^^8^

Breast massage and implant displacement techniques are postoperative methods that have been recommended to prevent the formation of capsular contracture despite limited evidence. Some surgeons recommend displacing the implant to maintain the breast pocket, whereas others use compression to flatten the implant and increase its surface area.^3^ Breast massage has been hypothesized to prevent breast contracture by disrupting postoperative capsular formation and effecting an anti-inflammatory displacement.^9^ More specifically, Vinnik's^2^ maneuvers were intended to expand the capsule anteriorly and toward the insertion of the *pectoralis*, which is where contracture often first begins; maneuvers for inferior expansion were not required, due to gravity's effect on the breast while standing. Riddle^4^ suggests that massage technique is more important than duration of exercises practiced; pressure should be exerted on the breast centrally and in all clockwise positions to displace the breast and maintain maximum surface area of the fibrous capsule.

Conversely, some skeptics believe breast massage may increase the rate of capsular contracture by increasing fibroblast activity and inflammation. Literature regarding the increased susceptibility to contracture with implant massage and displacement techniques is lacking. Nonetheless, a survey by Hidalgo and Sinno^10^ conducted in April 2015 revealed that 61.5% of the 1067 responding plastic surgeons still recommend postoperative massage.

Before comparing breast contracture rates, it is important to understand that intraoperative decisions regarding surgical technique and prosthesis choice will influence contracture outcomes. The IMF incision has the lowest risk of capsular contracture compared with other surgical routes in breast augmentation, postulated to be due to less bacterial contamination from the mammary ducts.^7^^,^^11^ A smooth breast implant surface, as opposed to a textured implant surface, may result in less bacterial adherence and subsequent breast contracture.^12^ However, a more recent meta-analysis showed that textured breast implants result in lower contracture rates.^6^ Finally, pocket plane may be another potentiator of capsular contracture—subglandular implants have an increased rate of capsular contracture compared with submuscular and subfascial implants, most likely due to the additional tissue layers separating the implant from glandular breast tissue.^6^

While the studies by Vinnik^2^ and Hipps et al^3^ were the most similar in surgical technique, implant prosthesis, and massage technique, they yielded opposite results regarding the efficacy of capsule expansion exercises in reducing capsular contracture rate. Vinnik^2^ reported a lower incidence of capsular contracture with exercises (28% vs 58%), whereas Hipps et al^3^ reported a higher capsular contracture rate (35% vs 30%). However, Vinnik^2^ combined capsule expansion exercises with triamcinolone use and did not evaluate the efficacy of capsule expansion exercises alone. Hipps et al^3^ also instilled steroids intraoperatively, but the data do not specify which cohorts of patients received steroids alone, massage alone, or both. Since it is known that steroids decrease the rate of capsular contracture, one possible explanation is that capsule expansion exercises may augment the effects of triamcinolone. Both Vinnik^2^ and Hipps et al^3^ began the exercises 2 weeks postoperatively, although capsule formation may begin as early as postoperative day 3.^5^

The results of the other studies mentioned are more difficult to interpret and compare due to variations in exercise technique, frequency, and onset. Becker and Prysi^5^ did not describe the exercise in detail, whereas the studies by Vinnik,^2^ Hipps et al,^3^ and Riddle^4^ all employed daily capsule expansion exercises involving 3 maneuvers performed twice daily. Vinnik^2^ and Hipps et al^3^ started these exercises 2 weeks postoperatively, whereas Riddle^4^ and Becker and Prysi^5^ started the massages at postoperative days 2 and 3, respectively. The exercises Riddle^4^ prescribed were more involved than the Vinnik's^2^ exercises; these exercises involved multiple maneuvers that worked clockwise around the entire breast, as opposed to Vinnik's^2^ exercises, which consisted of 3 maneuvers total. Riddle^4^ also studied a smaller number of patients (40 total subjects) compared with Vinnik^2^ (80 total subjects) and Hipps et al^3^ (453 total subjects), which may have contributed to the difference in capsular contracture rate.

Other variations of breast massage exercises exist in the literature. Barker and Schultz^13^ described once-daily exercises with 3 maneuvers—pushing the breast capsule into the upper cavity, against the lateral wall, and against the medial wall for 3 minutes each. Barker and Schultz^13^ added an additional exercise, which consisted of the patient lying on her breasts on the floor for a period of 30 minutes daily. Barker and Schultz^13^ did not address the timing of massage onset after surgery or the total number of patients evaluated but reported a reduced percentage of capsular contracture from 35% to 5% in uncomplicated breast augmentation cases.

In addition to the existing differences in breast massage, patient compliance is another variable to consider. Of the 4 studies, only Riddle^4^ reported all subjects following the exercise regimen daily, although they did not time the exercise period for the full 5 minutes.

Ultimately, massage technique and frequency are qualitative and subject to physician preference. In a more recent article, Becker and Springer^12^ reported a decreased incidence of capsular contracture from 20% to less than 2% in their breast augmentation cases over the last 15 years. Becker and Prysi's^5^ updated postoperative recommendations include early implant movement (a more disruptive technique than breast “massage” exercises) 3 times daily starting within 2 days of surgery, rather than 2 weeks; the movements should be performed more aggressively if firmness of the breast was noticed. Conversely, Burkhardt^14^ no longer recommends postaugmentation breast massage due to the lack of biological and clinical evidence for its efficacy; developing capsular contracture after implementing breast massage exercises often falsely and unnecessarily causes the patients to feel as they are at fault. More than half of surgeons may recommend breast massage exercises after breast augmentation, but significant variation of timing, onset, and technique exists.^10^

## CONCLUSIONS

While many techniques have been proposed and practiced in the prevention of capsular contracture, breast massage and implant displacement technique remains an area of controversy. A method to quantify adequate breast massage has been proposed, but the exercises still vary in technique, timing, and frequency of implementation after surgery. Among the articles that have analyzed incidence of capsular contracture after breast massage and displacement, the results regarding the efficacy of these exercises is conflicting. On the basis of the available data, breast massage has *not* been shown to decrease the formation of capsular contracture.

## Figures and Tables

**Table 1:** Characteristics of cited studies*

Study	Cases	Surgical technique	Implant type	Massage technique	Follow-up	Contracture rate with exercises	Contracture rate without exercises
Vinnik^2^	82	IMF incision, 40 mg of triamcinolone instilled in each pocket	Low-profile, round, gel-filled prosthesis without fixation patches	Capsule expansion exercises	12-36 mo	29% (11/38)^†^	68% (30/44)
Hipps et al^3^	453	IMF incision, subglandular insertion	Smooth-surfaced, soft, gel-filled prosthesis without fixation patches	Capsule expansion exercises	1 wk, 2 wk, monthly for 3 mo, every 3 mo for 1 y	35% (85/243)	30% (62/210)
Riddle^4^	40	Subpectoral insertion; each outer lumen filled with 20 mg of Solu-Medrol (methylprednisolone sodium succinate), 500 mg of Ancef (cefazolin), and normal saline	Double-lumen gel-filled prosthesis	Capsule expansion exercises	1 wk, 2 wk, 1 mo, 3 mo, and 6 mo	0% (0/20)	90% (18/20)
Becker and Prysi^5^	12	IMF, periareolar, or axillary incisions, breast pocket irrigated with bacitracin	Expander implant	Breast massage	Not specified	8% (1/12)	N/A

*IMF indicates inframammary fold; N/A, not available.

†Vinnik utilized 40 mg of triamcinolone in conjunction with postoperative breast exercises. There was no group studying the effects of postoperative breast exercises alone.

**Table 2:** Comparison of breast massage techniques

Study	Technique	Frequency	Onset of massage
Vinnik^2^	Supine, 3 maneuvers during which the breast was pressed firmly against the capsule in a uniform fashion for 15 s, 2 min total for both breasts	Twice daily	Postoperative week 2
Hipps et al^3^	Same technique as Vinnik	Twice daily	Postoperative week 2
Riddle^4^	Supine position, multiple clockwise maneuvers exerting as much force as tolerated, 5 min per breast	Twice daily	Postoperative day 3
Becker and Prysi^5^	Applied 80-300 mm Hg of pressure	Not specified	Postoperative day 2
